# Assessment of knowledge, attitudes, and practices towards Zika virus among healthcare workers in St. Kitts

**DOI:** 10.1186/s12879-021-05932-z

**Published:** 2021-03-05

**Authors:** Donya L. Francis, Utoomporn Wongsin, Shuo-Chen Chien, Yi-Hsin ( Elsa) Hsu, Franziska Michaela Lohmeyer, Wen-Shan Jian, Li-Fong Lin, Usman Iqbal

**Affiliations:** 1 Global Health & Development Department, College of Public Health, Taipei Medical University, No. 172-1, Sec. 2, Keelung Rd, Daan District, Taipei City, 106 Taiwan; 2 Global Health & Health Security Dept., College of Public Health, Taipei Medical University, Taipei, Taiwan; 3 Graduate Institute of Biomedical Informatics, College of Medical Science and Technology, Taipei Medical University, Taipei, Taiwan; 4 School of Gerontology Health Management, College of Nursing, Taipei Medical University, Taipei, Taiwan; 5 Executive Master Program of Business Administration in Biotechnology, College of Management, Taipei Medical University, Taipei, Taiwan; 6 School of Health Care Administration, College of Management, Taipei Medical University, Taipei, Taiwan; 7 Fondazione Policlinico Universitario A. Gemelli IRCCS, Rome, Italy; 8 Research Center for Artificial Intelligence in Medicine, Taipei Medical University, Taipei, Taiwan; 9 International Center for Health Information Technology (ICHIT), Taipei Medical University, Taipei, Taiwan; 10 Department of Physical Medicine and Rehabilitation, Shuang Ho Hospital, Taipei Medical University, New Taipei City, Taiwan; 11 Neuroscience Research Center, Taipei Medical University, Taipei, Taiwan; 12 Research Center for Artificial Intelligence in Medicine, Taipei Medical University, Taipei, Taiwan

**Keywords:** Global health, Public health, Zika virus, Healthcare workers, Knowledge, Attitudes, Practices, KAP, St. Kitts

## Abstract

**Background:**

Healthcare workers are usually the first responders during outbreaks and are instrumental in educating the populace about the prevention of different diseases and illnesses. The aim of this study was to assess the association between healthcare workers’ characteristics and knowledge, attitudes and practices toward Zika virus.

**Methods:**

This was a cross-sectional study that collected data from healthcare workers at 3 medical facilities using a validated self-administered questionnaire between July 2017 – September 2017. Logistic regression models were used to examine the association between sociodemographic and knowledge, attitudes, and practices.

**Results:**

A total of 190 healthcare workers were analyzed. Of these, 60, 72.6 and 64.7% had good knowledge, positive attitudes, and good practices toward Zika virus, respectively. Healthcare workers without a formal degree were less likely to have good knowledge of Zika virus (adjusted odds ratio (AOR) = 0:49; 95% confidence interval (CI) = 0.24–0.99) compared to those with a formal degree. Reduced odds for positive attitude towards Zika virus were observed in healthcare workers with low income as compared to those with high income (AOR = 0.31; 95% CI =0.13–0.75). Being younger than 40 years old was associated with poor Zika virus practices (AOR = 0:34; 95% CI = 0.15–0.79).

**Conclusions:**

Significant association between healthcare workers’ sociodemographic characteristics and Zika virus knowledge, attitudes and practices were observed. Public health interventions that seek to increase Zika virus awareness should aim to train healthcare workers who are younger, without formal degree and those earning low income.

## Background

Zika virus (ZIKV) is a major public health emergency with approximately 70 countries affected globally since 2015 [1]. ZIKV is a highly contagious mosquito-borne disease, which was first identified in 1947 among monkeys in Uganda, Africa [2]. Later, in early 1950s, the disease was observed in humans in Uganda and the Republic of Tanzania and has ever since posed a serious threat to humans especially pregnant women [2]. The virus is primarily transmitted by the *Aedes aegypti*, which is also responsible for the transmission of other similar mosquito-borne diseases such as dengue fever, and chikungunya [3]. The possibility of sexual transmission of ZIKV has also been reported [2].

The public health threat of ZIKV cannot be overlooked. During pregnancy, ZIKV is responsible for congenital brain abnormalities, including microcephaly and also triggers Guillain-Barré syndrome [2, 4]. For instance, in Brazil, ZIKV was linked to approximately 4000 microcephaly cases [5]. Considering the adverse health effects associated with ZIKV, the world health organization (WHO) outlined a Zika Strategic Response Framework which among others seeks to; enhance surveillance of ZIKV, and potential complications and Strengthen capacity in risk communication to engage communities to better understand risks associated with ZIKV [2]. Therefore, knowledge, attitude and practices of healthcare workers is crucial to achieving effective communication to the communities to improve the understanding of the associated risks.

Numerous studies have assessed the knowledge, attitude and practices (KAP) of ZIKV among the general population [6–9]. These studies have reported low knowledge in terms of ZIKV and therefore highlights the need to understand whether the same problem would be observed among healthcare workers. Healthcare workers are the main outlets of health information to the communities. As such, the KAP of healthcare workers toward ZIKV is vital in the efforts of improving awareness of the disease. Only few studies have sought to understand healthcare workers KAP toward ZIKV [10, 11]. In Indonesia, occupation, and type of work place for the healthcare workers were associated with good knowledge of ZIKV [10].

The first ever case of ZIKV in St. Kitts and Nevis was recorded in 2016 [12]. Ever since, St. Kitts and Nevis recorded an average of 16 cases per epidemiological week [12]. However, limited studies have examined ZIKV knowledge among healthcare workers in St. Kitts and Nevis, let alone, among the general population. Considering that ZIKV is an emerging public health issue, there is need to understand the healthcare providers’ KAP in terms of ZIKV. Therefore, this study aimed at assessing the KAP for healthcare workers from 3 medical facilities in St. Kitts and Nevis.

## Methods

### Study design and participants

This was a cross-sectional study that included healthcare workers from 3 medical facilities in St. Kitts and Nevis namely, Joseph Nathaniel France General, Pogson Medical Centre, and Department of Environmental Health.

### Sampling design and data collection

The study employed a convenient and purposive sampling method. Using an online sample calculator, “Raosoft.com”, a total of 189 participants was needed. The following parameters were used to determine the sample size: a response distribution of 50, 95% confidence level.

Data was collected through a self-administering technique using a structured questionnaire, which was adapted from a previous study [13]. A pilot study was conducted between May and June 2017. The questionnaire was divided into five sections. The first section asked collected sociodemographic information. The second section assessed the knowledge of the participants towards ZIKV. This section was made up of 15 yes/no questions. In the third section, a total of 10 questions were asked to assess the attitudes of the participants. Participants were asked yes/no and multiple-answer questions. These items recorded the participants’ beliefs and concerns about ZIKV and their ability initiate appropriate help-seeking behavior. The fourth section had a total of 15 questions on preventive practices. These items recorded the participants’ beliefs and concerns about ZIKV and their ability initiate appropriate help-seeking behavior. The questionnaire also contained two standalone questions. The first question was used to check if the participants had ever encountered a person suspected or confirmed as having ZIKV while the other asked about their sources of information.

### Inclusion and exclusion criteria

Participants in this study were included based on the following criteria: The availability and those who were employed in health-related positions or play a pivotal role in dealing with patients. Therefore, physicians, nurses, emergency medical technicians, pharmacists, lab technicians, radiologists, orderlies, dentists, secretaries, maids, environmental health workers and vector control officers were included. At the time of study medical/health-related students were present (working) at the sites, so they were included. Workers of non-health related posts; maintenance workers, auxiliary workers were excluded. Healthcare workers who were also absent during the time June–September 2017 were not included.

### Outcome measures

The main outcome of this study was the knowledge, attitude, and practices towards ZIKV. Knowledge, attitude, and practices were accessed as binary outcomes, respectively. The scores from the answers to the questions (see appendix-questionnaire) were created. The median was used as a cut-off point. Participants with scores above the median were regarded as having good knowledge, positive attitude, and good practices, respectively.

### Independent variables

A number of sociodemographic factors were considered in this study and these include age was analysed as (< 40 and ≥ 40), and gender (female and males). Marital status was analysed as a binary variable (married vs unmarried) with those single, widowed or divorced categorized as unmarried while, education was categorized as without degree (those with primary school, high school diploma and profession certification) and with degree (those with associate degree, university degree [bachelor or master], doctor of medicine and PhD). In terms of profession, the participants were categorized as Clinician (physicians and nurses) vs Non-Clinician (environmental health workers, secretaries, maids, environmental health workers and vector control). Place of employment was categorized as urban (JNF and Department of Environmental Health) and rural (Pogson Medical Hospital) while work experience was categorized as ≤5 and > 5 years. Finally, income was categorized as low (those earning less than or 3000 East Caribbean dollar) and high income (those earning more than 3000 Eastern Caribbean dollar).

### Statistical analysis

The data was analyzed using the Statistical Package for Social Sciences (SPSS) version 20. All statistical tests were two sided and the significance level was set at *p* < 0.05. Descriptive statistics of the sociodemographic factors and health factors were presented using number (n), and percentage (%). Chi-square tests was used to compare the differences in the distribution between those with good or poor knowledge, attitudes and practices. Logistic regression analysis was used to examine the association between the participants’ characteristics and knowledge, attitudes and practices towards ZIKV. Odds ratios (ORs) with their 95% confidence intervals (CIs) and *p*-values were reported.

## Results

A total of 190 healthcare workers were analyzed and 60% had good knowledge, 72.6% had positive attitudes, and 64.7% had good practices.

### Distribution of participants’ characteristics according to knowledge, attitude and practices

Table 1 presents the distribution of participants’ characteristics according to knowledge, attitudes and practices. There was a significance difference between those with poor and good knowledge of ZIKV in terms of education and income (*p* < 0.05) (i.e. a high proportion of those with a degree had good knowledge of ZIKV when compared to those without a degree 67.5% vs 32.5%). All other variables revealed insignificance in terms of knowledge.

**Table 1:** **Distribution of participants according to knowledge, attitude and practices** ***n*** **= 190**

	Knowledge			Attitude			Practice		
Variable	Poor n (%)	Good n (%)	p-value^**a**^	Poor n (%)	Good n (%)	p-value^**a**^	Poor n (%)	Good n (%)	p-value^**a**^
**Age**			0.290			0.295			0.020*
< 40	53 (69.7)	71 (62.3)		37 (71.2)	87 (63.0)		51 (76.1)	73 (59.3)	
≥ 40	23 (30.3)	43 (37.7)		15 (28.8)	51 (37.0)		16 (23.9)	50 (40.7)	
**Gender**			0.829			0.022*			0.866
Female	59 (77.6)	90 (78.9)		35 (67.3)	114 (82.6)		53 (79.1)	96 (78.0)	
Male	17 (22.4)	24 (21.1)		17 (32.7)	24 (17.4)		14 (20.9)	27 (22.0)	
**Marital status**			0.947			0.585			0.567
Married	21 (27.6)	32 (28.1)		13 (25.0)	40 (29.0)		17 (25.4)	36 (29.3)	
Unmarried	55 (72.4)	82 (71.9)		39 (75.0)	98 (71.0)		50 (74.6)	87 (70.7)	
**Education**			0.009**			0.288			0.577
Without degree	39 (51.3)	37 (32.5)		24 (46.2)	52 (37.7)		25 (37.3)	51 (41.5)	
With degree	37 (48.7)	77 (67.5)		28 (53.8)	86 (62.3)		42 (62.7)	72 (58.5)	
**Income**			0.007**			0.121			0.219
Low	51 (67.1)	54 (47.4)		24 (46.2)	81 (58.7)		33 (49.3)	72 (58.5)	
High	25 (32.9)	60 (52.6)		28 (53.8)	57 (41.3)		34 (50.7)	51 (41.5)	
**Occupation**			0.159			0.598			0.119
Clinician	43 (56.6)	76 (66.7)		31 (59.6)	88 (63.8)		37 (55.2)	82 (66.7)	
Non-Clinician	33 (43.4)	38 (33.3)		21 (40.4)	50 (36.2)		30 (44.8)	41 (33.3)	
**Work experience**			0.545			0.248			0.630
≤ 5	32 (42.1)	43 (37.7)		24 (46.2)	51 (37.0)		28 (41.8)	47 (38.2)	
> 5	44 (57.9)	71 (62.3)		28 (53.8)	87 (63.0)		39 (58.2)	76 (61.8)	
**Place of employment**			0.351			0.517			0.273
Urban	63 (82.9)	100 (87.7)		46 (88.5)	117 (84.8)		60 (89.6)	103 (83.7)	
Rural	13 (17.1)	14 (12.3)		6 (11.5)	21 (15.2)		7 (10.4)	20 (16.3)	

*p value < 0.05, **p value < 0.01, *** p value < 0.001, ^a^ Pearson chi-square

In terms of attitudes, there was a significance difference in the participants only in terms of gender, with a high proportion of female participants having good knowledge of ZIKV as compared to male participants.

For practice, a significant difference was observed in terms of age with younger participants having a high proportion of poor practices.

### Association between participants’ characteristics and knowledge

Table 2 illustrates the factors associated with participants’ knowledge on ZIKV. In the univariate analysis, being without a formal degree (odds ratio (OR) =0.46; 95% confidence interval (CI) = 0.25–0.83) was significantly associated with reduced odds of having good knowledge of ZIKV as compared those with degrees. Having a low income (OR = 0.44; 95% CI = 0.24–0.81) was also significantly associated with reduced odds of having good knowledge of Zika. When adjusted for potential confounders in the multivariate analysis, those without a formal degree were still significantly less likely to have good knowledge of ZIKV (AOR 0:49; 95% CI 0.24–0.99). Having a low income (adjusted odds ratio (AOR) = 0.58; 95% CI = 0.28–1.23) was found to no longer be significant in the multivariate analysis. Other factors were not significantly associated with knowledge.

**Table 2:** Factors associated with Healthcare Workers’ Knowledge on Zika Virus: univariate and multivariate analyses. n = 190

Variable	OR (95% CI)	p-value	AOR (95% CI)	p-value
**Age**				
< 40	0.72 (0.39–1.33)	0.291	0.55 (0.25–120)	0.131
≥ 40	1.00		1.00	
**Gender**				0.868
Female	1.08 (0.54–2.18)	0.829	1.07 (0.50–2.29)	
Male	1.00		1.00	
**Marital status**				
Unmarried	0.98 (0.51–1.87)	0.947	1.08 (0.53–2.18)	0.838
Married	1.00		1.00	
**Education**				
Without Degree	0.46* (0.25–0.83)	**0.010**	0.49* (0.24–0.99)	**0.049**
With Degree	**1.00**		1.00	
**Income**				
Low	0.44** (0.24–0.81)	**0.008**	0.58 (0.28–1.23)	0.154
High	**1.00**		1.00	
**Occupation**				0.966
Clinician	1.54 (0.84–2.79)	0.160	1.02 (0.50–2.06)	
Non-Clinician	1.00		1.00	
**Work experience**				0.692
≤ 5	0.83 (0.46–1.51)	0.545	1.16 (0.55–2.44)	
> 5	1.00		1.00	
**Place of employment**		0.535		0.586
Rural	1.47 (0.65–3.34)		1.28 (0.53–3.08)	
Urban	1.00		1.00	

*p < 0.05, ** *p* < 0.01, OR-Odds ratio, AOR-Adjusted odds ratio, Logistic Regression

### Association between participants’ characteristics and attitudes

Table 3 displays the association of participants’ characteristics and attitudes towards ZIKV. In the univariate analysis, being female (OR = 2.31; 95% CI = 1.11–4.78) was significantly associated with increased odds of having good attitudes towards ZIKV as compared to being male. In the multivariate analysis, being female was not significantly associated with having good attitudes toward ZIKV (AOR = 1.83; 95% CI = 0.84–3.99) while having a low education (AOR = 0.40; 95% CI = 0.18–0.90), having low income (AOR = 0.31; 95% CI = 0.13–0.75) were significantly associated with reduced odds of having good attitudes on ZIKV.

**Table 3:** Univariate and Multivariate logistic regression association of sociodemographic factors and Attitudes on Zika Virus n = 190

Variable	OR (95% CI)	p-value	AOR (95% CI)	p-value
**Age**				
< 40	0.69 (0.35–1.38)	0.297	0.60 (0.25–1.43)	0.250
≥ 40	1.00		1.00	
**Gender**				
Female	2.31* (1.11–4.78)	**0.024**	1.83 (0.84–3.99)	0.128
Male	**1.00**		1.00	
**Marital Status**				
Unmarried	0.82 (0.39–1.69)	0.585	0.82 (0.38–1.79)	0.614
Married	1.00		1.00	
**Education**				
Without Degree	0.71 (0.37–1.34)	0.289	0.40* (0.18–0.90)	**0.026**
With Degree	1.00		1.00	
**Income**				
Low	0.60 (0.32–1.15)	0.123	0.31* (0.13–0.75)	**0.010**
High	1.00		1.00	
**Health-Work status**				0.322
Clinician	1.19 (0.62–2.29)	0.598	1.51 (0.67–3.40)	
Non-Clinician	1.00		1.00	
**Work experience**				0.498
≤ 5	0.68 (0.36–1.30)	0.249	0.75 (0.33–1.71)	
> 5	1.00		1.00	
**Place of employment**				0.592
Rural	1.38 (0.52–3.63)	0.519	0.75 (0.27–2.12)	
Urban	1.00		1.00	

*p < 0.05, OR- Odds ratio, AOR- Adjusted odds ratio, Logistic Regression

### Association between participants’ characteristics and practices

Table 4 shows the association of participants’ characteristics and practices on ZIKV. In the univariate analysis, being younger (< 40) was (OR = 0.45; 95% CI = 0.24–0.89) was significantly associated with reduced odds of having good practices towards of ZIKV as compared those who were older. Other characteristics were not significantly associated with practices. After adjusting for potential confounders, age remained significantly associated with reduced odds of having good practices, with younger participants (< 40) showing reduced odds (AOR = 0:34; 95% CI 0.15–0.79). Income and occupation also exhibited significant associations with practices. Having a low income (AOR = 0.36; 95% CI = 0.16–0.81) was associated with reduced odds of having better practices than those in high income bracket while being a clinician (AOR = 2.78; 95% CI = 1.29–5.99) showed increased odds of having better practices than their counterparts.

**Table 4:** Association of Healthcare Workers’ sociodemographic factors and Practices on Zika Virus: univariate and multivariate n = 190

Variable	OR (95% CI)	p-value	Practices AOR (95% CI)	p-value
**Age**				
< 40	0.45* (0.24–0.89)	**0.022**	0.34* (0.15–0.79)	**0.011**
≥ 40	**1.00**		**1.00**	
**Gender**				0.368
Female	0.94 (0.45–1.94)	0.866	0.70 (0.32–1.53)	
Male	1.00		1.00	
**Marital status**				
Unmarried	1.19 (0.65–2.19)	0.577	0.92 (0.44–1.90)	0.814
Married	1.00		1.00	
**Education**				
Without Degree	0.82 (0.42–1.61)	0.568	0.75 (0.36–1.58)	0.455
With Degree	1.00		1.00	
**Income**				
Low	1.69 (0.38–1.25)	0.220	0.36* (0.16–0.81)	**0.014**
High	1.00		**1.00**	
**Occupation**				
Clinician	1.62 (0.88–2.99)	0.121	2.78** (1.29–5.99)	**0.009**
Non-Clinician	1.00		**1.00**	
**Work experience**				
≤ 5	0.86 (0.47–1.58)	0.630	1.29 (0.60–2.76)	0.518
> 5	1.00		1.00	
**Place of employment**				0.307
Rural	0.60 (0.24–1.50)	0.277	0.60 (0.23–1.60)	
Urban	1.00		1.00	

*p < 0.05, **p < 0.01, OR-Odds ratio, AOR- Adjusted odds ratio, Logistic Regression

## Discussion

According to literature, this is the first study that sought to examine healthcare workers KAP towards ZIKV in St. Kitts and Nevis. This study demonstrated that sociodemographic are associated with knowledge, attitude and practices among healthcare workers in St. Kitts and Nevis. Specifically, clinicians had 2.78-fold increased odds of exhibiting better practices for ZIKV while healthcare workers without a degree had reduced odds of having good knowledge and positive attitudes toward ZIKV, respectively.

Compared to previous studies in [10, 14], healthcare workers were observed to have relatively high knowledge (60.2%), attitude (72.6%), and practices (64.7%). In Indonesia, only 35.9% of healthcare workers had good knowledge of ZIKV [10]. St Kitts and Nevis is a smaller country as compared to Indonesia. As such, information on new cases and emerging diseases may be easily passed to people including healthcare workers. This might explain the differences in the observed knowledge rates between the two countries.

The current study revealed that healthcare workers without formal degree were less likely to exhibit good knowledge and better practices towards ZIKV. This makes intuitive sense because education has long been associated with good health practices including uptake of health information [15]. A study conducted in the Middle East [16] revealed significant association between education and knowledge of ZIKV. Educated healthcare workers are more likely to live in the urban areas and have access to information as compared to non-educated healthcare workers and subsequently, this may impact on their knowledge and attitude towards ZIKV.

Low income earners were less likely to have better practices towards ZIKV. The influence of income or wealth on health cannot be overemphasized. Rich people are more likely to have access to information, as well as access better services such as education as compared to poor individuals [17]. Healthcare workers with a higher income are more likely to have access to media via newspapers, radio as well as the internet as compared to low income earners who may not afford some of these. In the end, those that earn high income may be exposed to a lot of information including ZIKV and this may impact on their practices.

Younger workers (< 40 years) were less likely to have good practices towards ZIKV. A study conducted by Cheema at al [16]. reported that older healthcare workers were more likely to have good knowledge about ZIKV. This good knowledge could in turn help healthcare workers to possess better practices of ZIKV. Further, older health workers are more likely to have the required experience in handling emerging diseases which could in turn help their perceptions towards diseases like ZIKV and in the end help them have good practices.

Compared to non-clinical health workers, clinical health workers were more likely to have good practices of ZIKV. This result highlights the need to engage non-clinical health workers to improve their understanding of the disease which might in turn improve their practices. Both clinical and non-clinical health workers are important in the fight against diseases and thus, it is crucial to raise awareness of ZIKV in both groups of workers. This would ensure that these healthcare workers effectively pass on the health messages to the communities thereby helping in combatting the disease.

There are several strengths related to this study. First, this study was able to access both clinical and non-clinical health care providers, hence was able to provide an overview and comparison between these two groups. Secondly, the study was conducted at 3 main medical facilities in St. Kitts and Nevis which allowed for inclusion of a wide range of professionals. This was a cross-section study and as such, we could not infer causality to the associations observed. Further, the study relied on self-administered questionnaire for information which might be prone to interviewee error as some may not clearly understand the questions.

In this Covid19 pandemic era, these findings may not be directly relevant however, we assume that this study still contributes the important findings for the clinical and non-clinical health workers because it highlights the need to engage non-clinical health workers for improve their understanding about infectious disease that could lead to improve their practices.

## Conclusion

Sociodemographic such as age, income and education are important factors associated with KAP of ZIKV among healthcare professionals in St. Kitts and Nevis. In addition, the differences in clinical and non-clinical health workers were observed specifically in terms of the practices towards ZIKV. Therefore, public health programs aiming at improving awareness of ZIKV should not only aim at targeting the communities. These programs should also target health care providers specifically those that are non-clinical, younger, without formal degree and with low income. If awareness is raised in these professionals, their knowledge may be translated into the communities in which they serve.

## Data Availability

The data used for this analysis is available from the corresponding author on reasonable request.

## Abbreviations

KAP: Knowledge, Attitudes and Practices
ZIKV: Zika Virus

## Declarations

All methods were carried out in accordance with relevant guidelines and regulations.
